# Correlation Between Gait Performance and Global Cognitive Functions in Senior Adults: A Systematic Review

**DOI:** 10.1002/alz70858_096645

**Published:** 2025-12-24

**Authors:** Joel Acevedo, Karen Martinez, Kun Liu, Claudia Amaya

**Affiliations:** ^1^ University of Puerto Rico, Medical Sciences Campus, San Juan, PR, Puerto Rico; ^2^ Yale University, Hartford, Ct, CT, USA

## Abstract

**Background:**

Gait Performance (GP) and Global Cognitive Functions (GCF) are both critical for maintaining independence and quality of life in senior adults. Recent studies have explored the potential link between GP and GCF, encompassing executive functions.

**Method:**

PRISMA guidelines will govern this systematic review. This systematic review synthesizes published research from 2000 to 2024, including peer‐reviewed articles, pilot studies, and randomized controlled trials, to examine the relationship between GP (how a person walks and stands) and GCP in older adults. The exclusion criteria will be based on studies focused on physical activities unrelated to balance, meta‐analysis, and systematic reviews and those published in languages other than English or Spanish.

**Result:**

Our preliminary data indicate that gait, or walking speed, is significantly correlated with GCP in older adults, with slower walking associated with poorer global cognition. Specifically, gait speed during Dual‐Task Walking shows a strong correlation with working memory (*p* < .001) and processing speed (*p* < .05) in individuals aged over 60. Gender differences were observed, with women over 80 walking slower than men over 85, who walked faster, and women exhibited poorer global cognition than men.

**Conclusion:**

Overall, a gait slowing between 0.68 and 1.1 meters per second could predict a marker for the risk of developing dementia, indicating that monitoring gait speed in older adults may provide early warning signs, allowing for timely interventions. Enhancing GP and GCF can improve the quality of life and independence in older adults.

